# Contribution to the Study of the Technic of the Interventions on Peripheral Nerves

**Published:** 1919

**Authors:** 


**Contribution to the Study of the Technic of the Interventions on Peripheral Nerves.** By Dr. Georges Debaisieux. *Bulletin de l'Ambulance de la Panne*, July, 1917.

The author reports his experience after 60 interventions on wounded nerves. All of these interventions were performed secondarily, after complete cicatrization of the wound. The operation consists of the search and discovery of the injured nerve, the treatment of the lesion, and the closing of the wound.

The author thinks it advisable to incise the teguments along the classical line of research of the nerve. He does not advocate the use of the missile's tract.

On incising along the normal line the incision generally crosses the cutaneous scar which can be circumscribed to allow of its excision.

The middle part of the incision corresponds to the supposed situation of the lesion.

Long incisions are very advisable. They ought to be several centimeters longer than the scar, since the research of the nerve on the level of the scar is particularly difficult. It is much more easy to search it in sound tissues, and free it there carefully on both extremities of the wound. After this freeing is obtained above and beneath the lesion, the author slips beneath it two rubber sheets, about 2 cm. wide, which allow handling of the nerve without injuring it.

The complete freeing of the nerve in the region of the scar is then easy. It should be done from the central to the distal portion so as to avoid more surely the cutting off of the collateral branches.

*Treatment of the Lesion.* According to the importance of the lesions and the severity of functional disturbances, the surgeon resorts to either simple freeing or partial or total resection.

If the nerve trunk is fairly sound and the lesion proceeds from some scar adhesion, or fibrous or callous compression, simple freeing is usually efficient.

In the worst cases, on the other hand, the nerve may be either completely destroyed in one point or changed into a dense fibrous cord. Regeneration of the peripheral end can only take place after complete removal of all the injured part and direct or indirect suturing of both extremities. The author thinks that the careful touching of the nerve between the thumb and first finger is the best way of determining the extent of the sclerous lesions and the limits of the resection.

The cutting off should be made transversally with a very sharp and thin blade.

The important thing while suturing both ends is to have regard for the localization of the sensory and motary fibres in the central and distal portions of the nerve. There must be no twisting of one of the extremities.

If the loss of substance after resection does not overreach 3 or 4 cm., direct suturing is generally possible. A slight traction exerted on the central end, and the placing of the limb in a special position, will usually allow of direct suturing. This is best obtained by a circular row of stitches passing through the neurilemma ; 6 to 8 stitches generally secure a perfect suturing ; knots must be kept rather loose.

*Suturing of the Wound.* The closing of the wound requires accurate hemostasis, insulation of the nerve, and careful restoration of the anatomical layers.

Complete hemostasis of the operatory wound is usually difficult to secure. The author has, for his own sake, given up the use of Esmarch's tube. The nerve's own small artery bleeds a little after the resection. If this hemorrhage does not stop spontaneously after a few minutes, this result is easily obtained by twisting the vessel slightly with a forceps.

When the hemostasis is secured, the author sweeps away all remaining clots of blood by washing the wound with a current of warm normal saline solution.

Next to this the nerve must be carefully embedded to keep it from contracting new adhesions. The best method is to bury it amid sound muscles; if this is impossible the nerve can be sheathed in a fatty layer removed from the anterior part of the abdominal wall.

If none of these procedures can be resorted to, Carrion's resorbable organic membranes may prove useful. They are well tolerated and secure a careful sheathing of the nerve.

Anatomical layers should be restored as well as possible, all sclerous tissues capable of exerting some direct or indirect compression on the nerve being completely removed.

